# A Mathematical Model for Dental Caries: A Coupled Dissolution-Diffusion Process

**DOI:** 10.6028/jres.096.035

**Published:** 1991

**Authors:** T. M. Gregory, L. C. Chow, C. M. Carey

**Affiliations:** American Dental Association Health Foundation, Paffenbarger Research Center, National Institute of Standards and Technology, Gaithersburg, MD 20899

**Keywords:** Ca flux, computer simulation, coupled dissolution-diffusion, dental caries, hydroxyapatite, P flux, permselective diffusion

## Abstract

Demineralization of tooth mineral in the caries process was studied using a computer model that simulates a diffusion controlled dissolution process. The model consists of a two-compartment system. An acidic solution in the outer (“plaque”) compartment was assumed to be large in volume so that its composition remained constant during the process. The solution in the inner (“lesion”) compartment was in equilibrium with the tooth mineral, but its composition changed in response to diffusion of ions between the two solutions through an infinitely thin barrier. The permselectivity of the diffusion barrier to cations and anions can be modified as desired thus allowing the effects of membrane on the diffusion-dissolution process to be examined. Because the losses of calcium (Ca) and phosphate (P) from the “lesion” to the “plaque” generally does not occur at a molar ratio of 5/3, the Ca to P ratio of the dissolving mineral, the composition of the “lesion” fluid can change significantly from the starting composition, and this in turn modifies the Ca and P fluxes. A steady state condition is eventually reached under which the ratio of flux of Ca to that of P becomes 5/3. The results of the simulation show that for a given “plaque” pH, the rate of demineralization at steady state was the highest for cation and the lowest for anion permselective membranes. These results were in good agreement with those from an experimental study under comparable conditions.

## 1. Introduction

**Figure 1:**
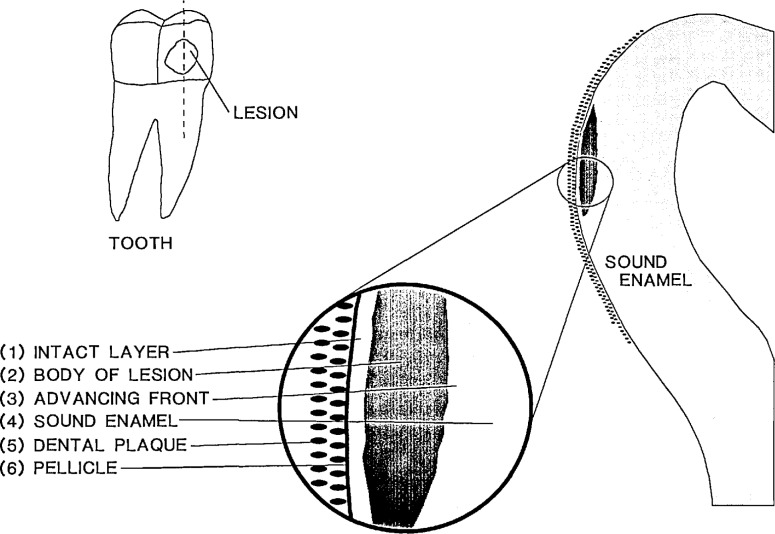
A schematic drawing of a typical dental caries lesion showing (1) an intact layer at the surface, (2) body of the lesion consisting of partially demineralized enamel, (3) the “advancing front” where active demineralization occurs, (4) sound enamel, (5) dental plaque covering the tooth, and (6) pellicle, adsorbed proteinaceous material on the tooth surface.

In the early stages of tooth decay, the affected tooth enamel usually is in the form of a subsurface lesion, Fig. 1. The outstanding features of a lesion are: (1) the “intact layer,” a layer of relatively sound enamel at the tooth surface, (2) the “body of the lesion” consisting of partially demineralized enamel, and (3) the “advancing front” where active demineralzation occurs. The subsurface demineralization of tooth in the caries process requires diffusion of acid ions into, and of solubilized mineral ions out of the lesion. Even when highly simplified, it is a relatively complex physicochemical process. Results from recent studies have shown that during the demineralization process, the solution within the lesion is approximately saturated with respect to the tooth mineral at all times [1,2]. These results indicate that the rate of dissolution of tooth mineral in the lesion is faster than the rate of transport of the mineral ions out of the lesion. Thus, the process of subsurface demineralization is diffusion controlled, and the rate of lesion progression may be expected to be strongly dependent on factors that govern the diffusion process.

Several mathematical models have been proposed to describe the diffusion process during lesion formation [3–7]. In the present study the caries process was also assumed to be controlled by the diffusion of ions, but it takes into consideration a most important phenomenon which has not been addressed in previous studies: the interaction between the permselective diffusion of ions and the dissolution of tooth mineral as described below.

The dissolution of tooth mineral at the advancing front (Fig. 1) adds calcium (Ca) and phosphate (P) ions to the solution within the lesion at a molar ratio of 5/3, the Ca/P ratio of the solid. On the other hand, the relative rates of the loss of Ca and P from the lesion to the plaque generally is not at a ratio of 5/3. This is because diffusion of the various Ca and P containing ions (Table 1) is largely controlled by factors such as the electrochemical potential gradients and the permselectivity of the diffusion barrier, which may consist of the body of the lesion, the intact layer, and the pellicle, etc. [8]. The unequal rates of addition and removal of Ca or P by the dissolution and diffusion processes, respectively, would lead to a change in the composition of the lesion fluid. This in turn modifies the driving forces for diffusion of all the ions. Thus, subsurface demineralization may be described as a coupled dissolution-diffusion process in which the composition of the saturated solution in the lesion changes in response to the diffusion of ions until a steady state is reached.

In previous studies [9–11] a diffusion cell comprising two compartments separated by an artificial membrane of known ion permselectivity was used as an experimental model to explore factors that may affect caries formation. One compartment (the “lesion”) contained an excess of hydroxyapatite (OHAp), Ca_5_(PO_4_)_3_OH, crystals, and its solution was kept at or near saturation by stirring. An undersaturated acidic calcium phosphate solution flowed continuously through the other compartment (the “plaque”), thus providing the driving force for dissolution of the crystals as modified by the permselectivity of the membrane. As described above, when the steady state is reached the composition of the “lesion” solution will become constant and the ratio of the flux of Ca to P will be 5/3. The results from the bench-scale caries model studies demonstrated that the composition of the saturated solution in the “lesion” can undergo great changes prior to attainment of steady state [9,10]. Furthermore, the large changes in the concentrations of the various ions in the “lesion” fluid, as affected by the permselectivity of the diffusion barrier, significantly increased or decreased the rate of mineral loss at steady state [11]. In the present paper we describe a mathematical model that simulates this bench-scale caries model so that the dynamics of diffusion-dissolution interactions may be more fully understood. The model also makes it possible to more rapidly survey the effects of a number of different variables on demineralization.

## 2. Methods

### 2.1 Overview

In this computer simulation of the caries-forming process, the transport of all solution species (Table 1) is tracked between a “plaque” compartment and a “lesion” compartment, separated by a barrier which blocks the free flow of fluids and is selectively permeable to all solution species. The “plaque” compartment is treated as a large reservoir of invariant composition with a fixed degree of under-saturation with respect to the dissolving mineral, OHAp. This composition can be selected to simulate various oral environments. The “lesion” compartment is visualized as a suspension of OHAp crystals maintained at or near saturation by dissolution or precipitation which is assumed to be more rapid than the diffusion process.

The computations proceed to convergence through a number of cycles, each consisting of two phases or loops. In the first phase (“diffusion loop”), diffusion across the barrier for a specified small time interval results in concentration changes in the “lesion” compartment. In the second phase (“dissolution loop”), saturation of the “lesion” solution is re-established. When the system has converged to a steady state, the composition of the “lesion” compartment becomes invariant with time, and concomitantly the molar ratio of the total flux of calcium *J*(Ca)_T_ to the total flux of phosphate *J*(P)_T_ attains the value 5/3, the Ca/P of the dissolving solid.

The model approximates continuous diffusion across an infinitely thin barrier separating the “lesion” from the “plaque.” Under this restriction the diffusion potential (Δ*E*) and the fluxes (*J*_s_) of the individual solution species are approximated [14] by the following equations:

$$\Delta E=-\left(RT/F\right)\sum_{s}\left(t_{s}/z_{s}\right)\ln\left(a_{s}^{L}/a_{s}^{P}\right)$$ (1)

and

$$J_{s}=-D_{s}C_{s}\left[\ln\left(a_{s}^{L}/a_{s}^{P}\right)+z_{s}\left(F/RT\right)\Delta E\right]$$ (2)

where

*T* = Temperature (K)
*R* =The gas constant (8.314 × 10^7^ J·mol^−1^·K^−1^)
*F* = Faraday constant (9.649 × 10^11^ J·V^−1^·mol^−1^)

and for the sth species, with the compartment designated by superscript,

*t*_s_ = transference number
*z*_s_ = valence
*D_s_* = diffusion coefficient within the barrier (cm^2^/s)
C_s_ = $\left(C_{s}^{L}+C_{s}^{P}\right)/2\left(\mathrm{mol}/L\right);$ the average concentration across the membrane
*a*_s_ = thermodynamic activity (mol/L).

It may be observed that the flux, *J*_s_, of any charged species is influenced by both chemical and electrical (membrane) potentials as indicated by the two terms in Eq. (2).

### 2.2 Characterization of the Barrier

For the barrier to exhibit the permselectivity of tooth enamel to individual ions, the diffusion contants, $D_{s}^{0},$ can be modified by arbitrary scaling factors. For example, to simulate an anion permselective membrane, the diffusion constants of all cations may be reduced by a common scale factor, *f*, using the equation,

$$D_{s}=f^{\left|z_{s}\right|}\cdot D_{s}^{0}$$ (3)

where $D_{s}^{0}$ is the diffusion coefficient of species s in water (Table 1). $D_{s}^{0}$ for the ions was calculated from the ionic mobility (μ_s_) using the Nernst-Einstein relation $\left[D_{s}^{0}=\left(RT/F\right)\mu_{s}\right]$ and for ion pairs, the Nernst method for the average diffusion coefficient [12]. Values of *f* of 1.0 (nonselective membrane) or 0.01 for either cations (anion permselective membrane) or anions (cation permselective membranes) were used in this study. The diffusion coefficients for H^+^ and OH^−^ were allowed to be invariant to account for the Grotthuss mechanism of H^+^ and OH^−^ association with the solvent (H_2_O) across the membrane [12].

#### 2.2.1 Input and Initial conditions

**Figure 2:**
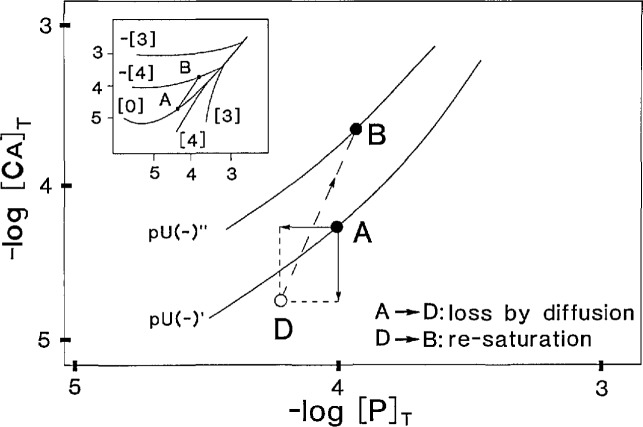
A schematic representation of the changes in the composition of the “lesion” solution, expressed in a −log[P]r vs −log[Ca]_T_ phase diagram, during a computation cycle. See Appendix for the definition of U.

1. All constants in the preceding list from *R* (gas constant) to $D_{s}^{0}$ (diffusion coefficients) were stored as functions of temperature in the program. Also stored were dissociation and association constants of all partially dissociated species such as weak acids, ion pairs, etc.
2. The temperature, *T*, was input for each run and was the same for both the “lesion” and “plaque” compartments.
3. For the “plaque” compartment, the composition of the solution was chosen to represent a fixed degree of undersaturation with respect to tooth mineral, OHAp, at the given temperature. This solution retained these initial concentrations.
  Table 2 lists the compositions of five “plaque-saliva” compositions that had been used in the previous experimental study and were used in the present simulation study. The solutions all contained 1 mmol/L HCl, 30 mmol/L KCl, with various amounts OHAp dissolved such that the pH of the solution ranged from 3.2 to 5.0 and $pIAP_{\mathrm{OHA}p}$ (*IAP* = ion activity product) ranged from 82.55 to 65.1.
4. For the initial solution in the “lesion” compartment, the composition was chosen to be the “plaque” solution that was made saturated with respect to OHAp (Table 2). This solution can be represented as a point on the p*U*(−)′ isotherm, e.g., point A, in the phase diagram shown in Fig. 2. The generalized fourth component [*U*(±)] in the system is defined in Appendix A.
5. Compositions of solutions in both compartments were initially adjusted to assure electroneutrality by an iteration procedure that finds the appropriate [H^+^] value (see Sec. 2.2.2,1 below).

#### 2.2.2 Diffusion Loop of Calculation

Objective: Compute state of the system in the “lesion” after an interval of mass flow.

1. Concentrations and activities of all species *C*_s_ (except [H^+^]) listed in Table 1 were calculated by an iterative process [15,16] using known equilibrium constants and a suitable approximation (Debye-Hückel) for the activity coefficients [15]. All constants were determined at the temperature of interest and are given in Table 3 for 25 °C. The computations were done just once for the “plaque” compartment because this compartment is held at fixed concentrations.
2. Next the diffusion potential, *ΔE*, was calculated according to Eq. (1); *ΔE* is a linear function of the terms consisting of logarithmic ratios of the activities in the two compartments, $\ln\left(a_{s}^{L}/a_{s}^{P}\right),$ and the transference numbers as shown in Eq. 1.
3. Calculation of mass flows was conducted as follows.
  - The fluxes, *J*_s_, were computed using the modified Nernst-Planck flux equation [Eq. 2]; the approximations used in deriving this equation as well as Eq. (1) for the diffusion potential require that all the quantities remain constant over a certain small time interval, *Δt.* It was thus necessary to limit the magnitude of concentration changes by adjusting the size of *Δt.*
  - The material flows are *ΔC_s_=J*_s_*Δt*, and the resulting new concentrations are C_s_′ = *C*_s_ + Δ*C*_s_. These two quantities were used in the following constraints to assure that the changes in the state of the system were so small that the assumption in 3a holds.
    - |Δ*C*_s_|/*C*_s_ must lie between predetermined limits.
    - For the new system composition, |Δ*E*_new_−Δ*E*_old_| must not exceed a predetermined limit.
    If either (i) or (ii) was not satisfied, a new smaller Δ*t* was chosen and Δ*C*_s_, etc., were recalculated.
  - Using the new ion concentrations, *C*s′, new total concentrations of the various components resulting from the mass flow were calculated. The value of [Ca]_T_ for the calcium component is the sum of all calcium-containing species and similarly for P, K, Cl and other components. The system will now be at a point “D” in the phase diagram (Fig. 2), representing a condition of undersaturation.
  - The total flux for each component was calculated for later use to check steady state condition. In particular, for the Ca and P components:
    $$\begin{array}{l} J{\left(\mathrm{Ca}\right)}_{T}=J\left({\mathrm{Ca}}^{2+}\right)+J\left({\mathrm{CaH}}_{2}{\mathrm{PO}}_{4}^{+}\right)+\ldots \\ J{\left(P\right)}_{T}=J\left(H_{3}{\mathrm{PO}}_{4}\right)+\ldots +J\left({\mathrm{CaH}}_{2}{\mathrm{PO}}_{4}^{+}\right)+\ldots \end{array}$$

where *J*(Ca)_T_ and *J*(P)_T_ are the sums of the flux for all species containing Ca and P, respectively.

#### 2.2.3 Dissolution Loop of Calculation

The next stage of the calculation was to re-establish saturation in the “lesion” compartment. This feature is a crucial part of the model that has not been implemented successfully in previous literature reports.

Objective: Drive the solution in the “lesion” from the undersaturated state (point D, Fig. 2) reached at (Sec. 2.2.2,3c) to a new equilibrium (saturated) condition. The position in the new composition will then be at point B, lying on an isotherm p*U*(−)″ (Fig. 2).

Method: With the total phosphate concentration, [P]_t_, and [H^+^] as variables, a nonlinear least squares procedure [29,30] was used to satisfy the conditions of electroneutrality and saturation (Eqs. (4) and (5)), forcing congruent dissolution/precipitation, i.e., Δ[Ca]_T_ = (5/3)Δ[P]_T_.

$$\sum_{s}z_{s}C_{s}=E\to 0$$ (4)

$$pIAP_{\mathrm{OHAp}}-pKSP_{\mathrm{OHAp}}=S\to 0$$ (5)

where $IAP_{\mathrm{OHAp}}$ is the ionic activity product and $KSP_{\mathrm{OHAp}}$ is the solubility product for OHAp (Table 3).

The least squares procedure utilizes an iterative process to achieve convergence to a minimum where the sum of *E*^2^ *+ S*^2^ approaches zero. The resulting composition is represented by a new point, B, on an isotherm p*U*(−)″ (Fig. 2). The isotherm p*U*(−)″ is different from the isotherm p*U*(−)′ in the previous cycle because of the change in the value of “electroneutrality unbalance” [8]. This is caused by the diffusion of non-consumed anions (e.g., Cl^−^) and cations (e.g., K^+^) [10] that occurred during the time interval *Δt.* The composition at point B was then compared to the composition at the commencement of the preceding mass flow stage (Point “A” in the diagram). If B differed from A, the steady state had not been reached and the system returned to step 2 in Sec. 2.2.2 until convergence, i.e., the steady state was attained.

#### 2.2.4 Steady State

At steady state the difference between points “A” and “B” in Fig. 2 becomes negligible. This implies that mass changes due to diffusion are exactly compensated by mass changes due to dissolution/precipitation reactions. Under these conditions *J*(Ca)_T_/*J*(P)_T_=5/3. The total fluxes were calculated at step 3d in Sec. 2.2.2 and the ratio was monitored throughout successive iterations.

## 3. Results

The composition of the “lesion” solution and the fluxes of the various components were found to change significantly from the starting point to the steady state. The rates of demineralization, expressed in the units μg OHAp/cm^2^ min, calculated from the steady state (Ca)_T_ or the (P)_T_ flux at various “plaque” pH values are given in Table 4. It can be seen that for a given type of membrane the rate of demineralization increased as the “plaque” pH decreased. For a given “plaque” pH the rate of demineralization is the highest with the cation permselective membrane and is the lowest with the anion permselective membrane. Although the effects of membrane permselectivity on rate of demineralization were similar in trend at the various “plaque” pH values, the effects became progressively greater as the “plaque” pH was decreased. For example, at pH 5.0 the relative rates of demineralization for the three membranes were nonselective: cation: anion = 100: 241:1.5, whereas at pH 3.2 the relative rates were 100: 258: 0.064. For comparison purposes the experimental values of Chow and Brown [11] are also given. It can be seen that the effects of pH and membrane permselectivity on the demineralization rate are similar between the computer simulation and the bench-scale experiments. The simulation and the experimental flux values were found to be strongly correlated with the correlation coefficients being equal to 0.995, 0.996, and 0.999 for the nonselective, cationic permselective, and anionic permselective membrane systems, respectively. As shown in Table 4, the average ratios of the simulation and experimental flux values for the three groups are 29.0, 43.0, and 1.3, repectively. The ratio, 29.0, for the nonselective membrane system may be taken as a proportionality constant needed to account for the inherent differences in the two models. The higher ratio, 43.0, for the cationic permselective membrane system and the lower ratio, 1.3, for the anionic permselective system suggest that the simulation model magnified the effects of the membrane, probably due to the high degree of permselectivity assigned to the membranes (0.01). The effects of membrane permselectivity on the rate of demineralization shown in Table 4 are in fact a result of the significant changes in the composition of the “lesion” solution that occurred from the beginning of the process to the time when steady state was reached. For the purpose of illustrating the dynamic nature of the diffusion-dissolution interactions, results from the simulations with a “plaque” pH of 4.5 are described in greater detail below.

Figure 3a shows the [Ca]_T_, [P]_T_, [K]_T_, [Cl]_T_, and the pH of the “lesion” solution as a function of time for the system with the nonselective membrane. The fluxes of these components and the membrane potential during the same time period are shown in Fig. 3b. It is noted that although a total of seven phosphate-containing species (Table 1) were considered in the calculations, in the pH range used, $H_{2}{\mathrm{PO}}_{4}^{-}$ is the predominant phosphate species and its diffusion accounts for the bulk of the P flux. Similarly, the diffusion of the free Ca^2+^, K^+^, and Cl^−^ ions accounts for nearly all of the Ca, K and Cl fluxes, respectively. Consequently, the changes in the total concentrations and total fluxes shown in Figs. 3a and 3b can be adequately understood by examining the diffusion of the corresponding predominant ions. At the beginning of the process, driving forces existed for the inward diffusion of H^+^ ions and outward diffusion of Ca^2+^ and $H_{2}{\mathrm{PO}}_{4}^{-}$ ions. The diffusion of these ions produced a slight negative membrane potential (−0.07 mV), which induced a short burst of K^+^ and Cl^−^ diffusion in opposite directions (Fig. 3b) even though no concentration gradient existed for either ion initially. The K^+^ and Cl^−^ diffusion ceased as the concentrations of these ions in the “lesion” solution approached those prescribed by the membrane potential following the Nernst Equation [14]. In approaching the steady state, the [Ca]_T_ and [P]_T_ increased slightly, from 0.741 and 0.444 mmol/L to 0.855 and 0.513 mmol/L, respectively, and the pH decreased slightly, from 5.929 to 5.855. The [K]_T_ decreased slightly, from 30.00 to 29.95 mmol/L, and the [Cl]_T_ increased slightly from 31.00 to 31.11 mmol/L. The (Ca)_T_ and (P)_T_ fluxes increased steadily to 1.324 and 0.795 mmol/cm^2^ sec, respectively, when the steady state was reached.

Figure 4a shows the [Ca]_T_, [P]_T_, [K]_T_, [Cl]_T_, and the pH of the “lesion” solution as a function of time for the system with the cation permselective membrane. The fluxes of these components and the membrane potential during the same time period are shown in Fig. 4b. It is seen from Fig. 4b that the cation permselective nature of the diffusion barrier allowed Ca^2+^ ions to diffuse out of the “lesion” rapidly during the early period, i.e., the first 20 h of the process. Although the flux of H^+^ was not followed in the calculations, the presence of a negative membrane potential at this time period would indicate that the inward diffusion of H^+^ was in fact more rapid than the outward diffusion of Ca^2+^. The negative membrane potential brought about a burst of outward diffusion of K^+^, an ion which did not have a concentration gradient at the start. The membrane potential reached a minimum of −0.9 mV at approximately 50 h and started to increase steadily in approaching the steady state. The change in the sign of the membrane potential can be attributed to a decreased inward diffusion of H^+^ because the “lesion” pH has been reduced from 5.93 to 5.45, diminishing the driving force for proton diffusion. The change in the sign of the membrane potential led to a corresponding change in the direction of K^+^ diffusion. After the rearrangement of dominant driving forces in the initial stage of the process (the first 200 h), the fluxes and concentrations of all ions approached the steady state values asymptotically. When the steady state was reached, the [Ca]_T_ increased significantly from 0.741 to 1.417 mmol/L, the [P]_T_ increased substantially from 0.444 to 9.576 mmol/L, the [K]_T_ increased from 30.00 to 34.61 mmol/L, and the [Cl]_T_ decreased from 31.00 to 27.70 mmol/L. It is interesting to note that at steady state the [Ca]_T_/[P]_T_ ratio was 0.148, which is drastically reduced from the initial value of 1.67. The (Ca)_T_ and (P)_T_ fluxes at steady state were 3.719 and 2.164, nearly triple the corresponding fluxes in the system with the nonselective membrane.

Figure 5a shows the [Ca]_T_, [P]_T_, [K]_T_, [Cl]_T_, and the pH of the “lesion” solution as a function of time for the system with the anion permselective membrane. The fluxes of these components and the membrane potential during the same time period are shown in Fig. 5b. It is seen from Fig. 5b that during the initial period, e.g., the first 5 h, the anion permselective nature of the membrane allowed the P ions to diffuse out readily. This and the inward diffusion of H^+^ (as described in “Methods,” in the present model H^+^ ion transport was not impeded by the anion permselective membrane) produced a negative membrane potential. The negative potential prompted an inward diffusion of Cl^−^ ions which gradually diminished as the steady state was approached. The changes in concentrations of the various components were relatively small. At steady state the [Ca]_T_ increased slightly from 0.741 to 0.861 mmol/L, and the [P]_T_ decreased from 0.444 to 0.425 mmol/L, resulting in an increase in [Ca]_T_/[P]_T_ ratio from 1.67 to 2.03. The [K]_T_ decreased from 30.00 to 29.90 mmol/L, and [Cl]_T_ increased from 31.00 to 31.17 mmol/L. The fluxes of (Ca)_T_ and (P)_T_ were 0.00963 and 0.00665, respectively. These values are over 100 times smaller that those in the system with the nonselective membrane.

## 4. Discussion

The mathematical caries model described in the present study is a simulation of the experimental bench-scale caries model reported previously [9–11]. The model was aimed at examining the interactions between the diffusion and dissolution processes that occur during the caries progression. Because in this model the solution species were allowed to diffuse across an infinitely thin barrier separating the “lesion” from the “plaque,” the model is inherently limited in that it does not address certain characteristics of a true lesion such as the presence of concentration gradients within the lesion. The present model also does not describe the mechanism for the formation of the mineral dense surface layer. On the other hand, the model clearly demonstrated the effects of membrane permselectivity on lesion composition and rate of demineralization. The results of the simulations parallel closely those obtained in the bench-scale caries model in several ways as described below.

As described in Sec. 2, the compositions of the “plaque” solutions are such that they had the same HCl and KCl concentrations as those of the initial saturated solution in the “lesion” compartment but had lower amounts of OHAp dissolved (Table 3). Consequently, if one were to dissolve OHAp in any of the “plaque” solutions until saturation was reached, the solution would be the same as the initial “lesion” solution. The fact that in many cases the steady state [Ca]_T_ and/or [P]_T_ of the “lesion” solution became greater than the corresponding values in the initial “lesion” solution demonstrates the importance of the membrane effects on OHAp dissolution.

The [Ca]_T_/[P]_T_ ratio of the “lesion” solution at steady state was significantly lower than the initial ratio of 5/3 for the system with the cation-selective membrane and higher for the system with the anion-selective membrane [9,10]. Although the membrane potentials were relatively small due to the presence of a background electrolyte [10], i.e., 30 mmol/L of KCl, the redistribution of [K]_T_ and [Cl]_T_ in accordance with the membrane potential led to the significant change in [Ca]_T_/[P]_T_ as mentioned above.

For a given cariogenic challenge, i.e., a given “plaque” pH, the rate of demineralization is highly dependent on the membrane permselectivity (Table 4). It is worth noting that although the cationic permselective membrane reduces the diffusion of all anions, it actually led to significantly increased fluxes of Ca and P when compared to those obtained with the nonselective membrane. On the other hand, the anionic permselective membrane, which reduces the diffusion of all cations (except H^+^), decreased the fluxes by a hundred fold.

The above findings, which are in good agreement with the experimental data, are a direct consequence of the diffusion-dissolution interactions. Previously reported mathematical models for caries [3–7,31,32], although possibly more sophisticated in some respects, have not been able to demonstrate this important phenomenon that has been confirmed in both bench-scale [9–11] and microanalytical [1,2] caries models. One model [31,32] utilizes coupled diffusion as a basis for describing the effects of diffusion on dissolution. This model, which becomes mathematically complicated when the number of components is three or higher, was not developed to study the effects of membrane permselectivity on rate of demineralization.

In this initial assessment of the simulation model, the same “plaque” solutions as those employed in the bench-scale model were used. In future studies, “plaque” solutions with compositions that mimic more closely those of real plaque under cariogenic conditions should be used. The effect of weak acids such as lactic or acetic acid in the “plaque” compartment will differ from that of HCl because the weak acid will be only partially dissociated in the pH range associated with cariogenic conditions. The presence of fluoride in the “plaque” solution or the presence of fluorapatite (FAp) in the enamel “lesion” should produce significant effects on the composition of the lesion and the rate of demineralization [33].

Since the demineralization occurs primarily at the advancing front of the lesion, the demineralizing acid must be transported from the plaque through the diffusion barrier (the intact layer and the body of the lesion) to the advancing front in order to effect demineralization. Similarly, the solubilized ions, e.g., Ca^2+^, $H_{2}{\mathrm{PO}}_{4}^{-}$, etc., must be transported from the advancing front of the “lesion” towards the plaque. Thus, concentration gradients of all the diffusion species exist within the barrier. Although the present model is an approximation of the diffusion process taking place during subsurface demineralization, it clearly demonstrated the results of the interactions between the diffusion and dissolution processes. A model that allows for a diffusion barrier with a finite thickness such that concentration gradients of the diffusing species may exist within the barrier should further improve the ability to predict the effects of various factors on the caries process.

## Figures and Tables

**Figure 3a:**
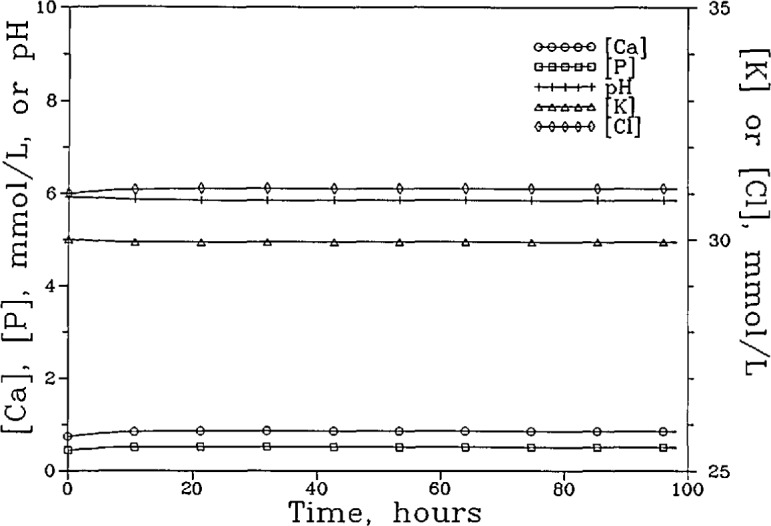
The [Ca]_T_, [P]_T_, [K]_T_, and [Cl]_T_ of the “lesion” solution as a function of time for the system with the nonpermselective membrane and a “plaque” pH of 4.5.

**Figure 3b:**
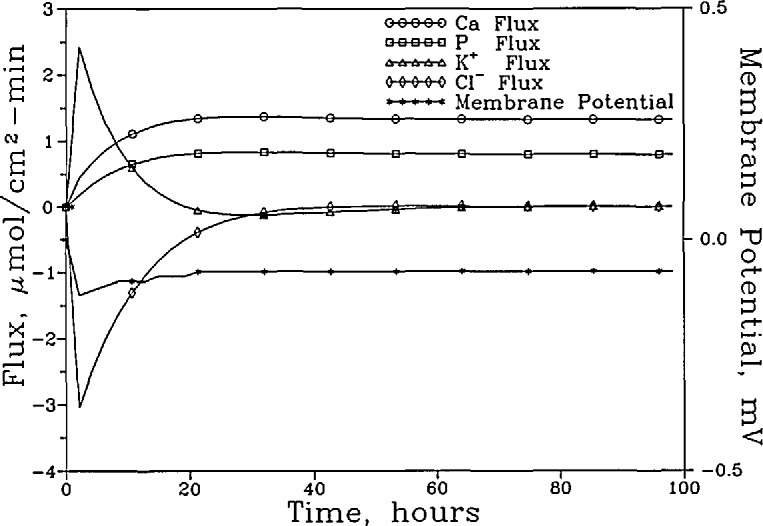
The fluxes of these components and the membrane potential over the same time period.

**Figure 4a:**
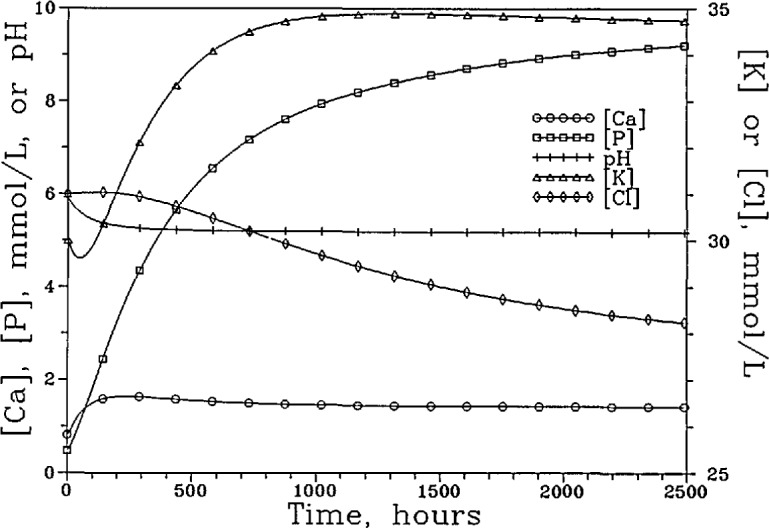
The [Ca]_T_, [P]_T_, [K]_T_, and [Cl]_T_ of the “lesion” solution as a function of time for the system with the cation-permselective membrane and a “plaque” pH of 4.5.

**Figure 4b:**
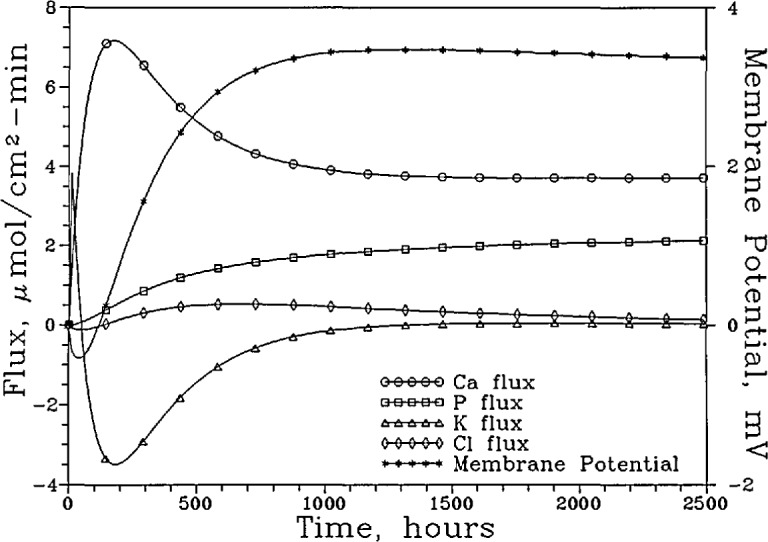
The fluxes of these components and the membrane potential over the same time period.

**Figure 5a:**
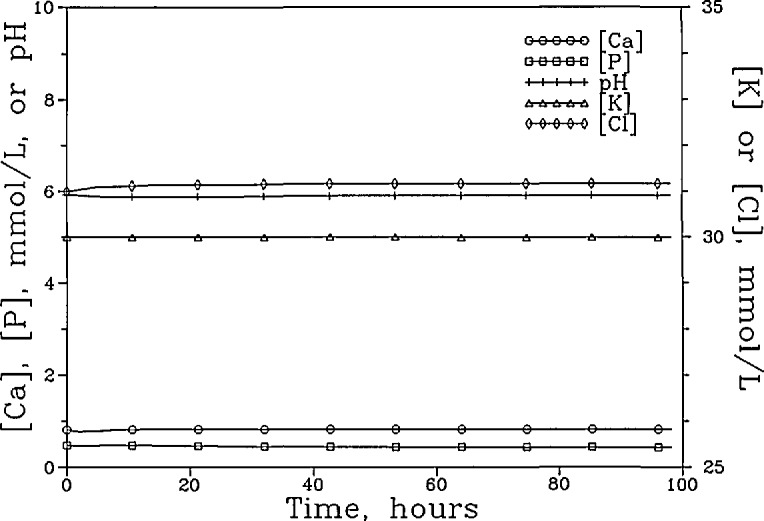
The [Ca]_T_, [P]_T_, [K]_T_, and [Cl]_T_ of the “lesion” solution as a function of time for the system with the anion-permselective membrane and a “plaque” pH of 4.5.

**Figure 5b:**
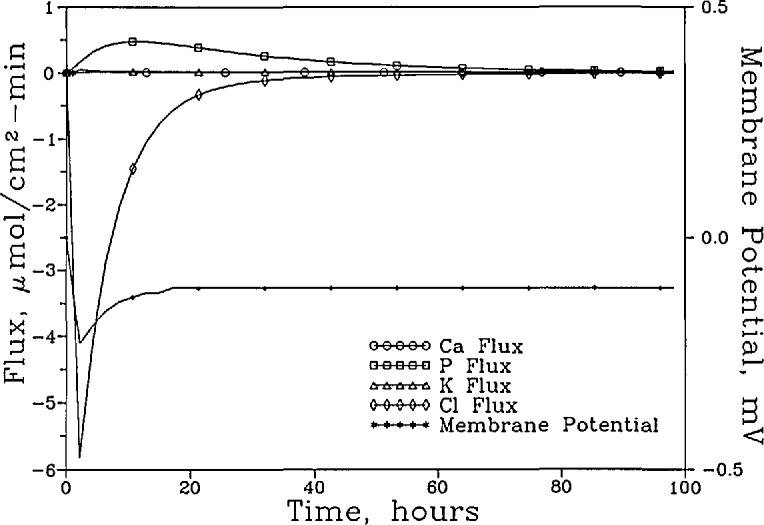
The fluxes of these components and the membrane potential over the same time period.

**Table 1:** Diffusing species and diffusion coefficients at 25 °C

Species	D^0^×10^5^ cm^2^/s	Species	D^0^×10^5^ cm^2^/s
H^+^	9.3^a^	OH^−^	5.28^a^
K^+^	1.96^a^	CI^−^	2.03^a^
Ca^2+^	0.79^a^	$H_{2}{\mathrm{PO}}_{4}^{-}$	0.88^b^
${\mathrm{CaH}}_{2}{\mathrm{PO}}_{4}^{+}$	0.62^c^	${\mathrm{CaPO}}_{4}^{-}$	0.29^c^
CaOH^+^	1.03^a^	${\mathrm{HPO}}_{4}^{2-}$	0.44^b^
H_3_PO_4_	1.61^b^	${\mathrm{PO}}_{4}^{3-}$	0.61^b^
CaHPO_4_	0.28^c^		

a $D_{s}^{0}$ calculated from values given in [12].

b $D_{s}^{0}$ calculated from values given in [13].

c $D_{s}^{0}$ calculated from values given in [12] and [13].

**Table 2:** Composition and $pIAP_{\mathrm{OHA}p}$ of the “plaque” and initial lesion solutions

	pH	Tot Ca mmol/L	Tot PO_4_ mmol/L	K mmol/L	CI mmol/L	p*IAP*_OHAp_^a^
Plaque solutions						
A	3.194	0.741	0.444	30.0	31.0	82.55
B	3.503	0.185	0.111	30.0	31.0	77.17
C	4.003	0.630	0.378	30.0	31.0	72.48
D	4.503	0.689	0.413	30.0	31.0	68.66
E	4.998	0.709	0.425	30.0	31.0	65.10
Initial lesion solution						
	5.929	0.741	0.444	30.0	31.0	58.55

a $pIAP_{\mathrm{OHA}p}$ values calculated at 25 °C, appropriate constants given in Table 3.

**Table 3:** Constants at 25 °C

Equilibrium constant (*K*)		

Reaction	*K*	Ref.
$K_{p1}:H_{3}{\mathrm{PO}}_{4}⇄H^{+}+H_{2}{\mathrm{PO}}_{4}^{-}$	7.11×10^−3^	[17]
$K_{p2}:H_{2}{\mathrm{PO}}_{4}^{-}⇄H^{+}+{\mathrm{HPO}}_{4}^{2-}$	6.31×10^−8^	[18]
$K_{p3}:{\mathrm{HPO}}_{4}^{2-}⇄H^{+}+{\mathrm{PO}}_{4}^{3-}$	4.52×10^−13^	[19]
*K_w_*: H_2_O⇆H^+^ + OH^−^	1.01×10^−14^	[20]
Debye (A)	0.5115	[21]

Association constant (*K*_a_)		

Ion pairs	K	Ref.

$K_{{\mathrm{CaH}}_{2}{\mathrm{PO}}_{4}^{+}}:{\mathrm{Ca}}^{2+}+H_{2}{\mathrm{PO}}_{4}^{-}⇄{\mathrm{CaH}}_{2}{\mathrm{PO}}_{4}^{+}$	8.45	[22]
$K_{{\mathrm{CaHPO}}_{4}^{0}}:{\mathrm{Ca}}^{2+}+{\mathrm{HPO}}_{4}^{2}⇄{\mathrm{CaHPO}}_{4}^{0}$	265.0	[22]
$K_{{\mathrm{CaPO}}_{4}^{-}}:{\mathrm{Ca}}^{2+}+{\mathrm{PO}}_{4}^{3-}⇄{\mathrm{CaPO}}_{4}^{-}$	2.90×107	[23]
$K_{{\mathrm{CaOH}}^{+}}:{\mathrm{Ca}}^{2+}+{\mathrm{OH}}^{-}⇄{\mathrm{CaOH}}^{+}$	20.0	[24]

Solubility product (*KSP*)		

Ca−PO4 salt	K	Ref.

KSPOHAP: Ca5(PO4)3OH	2.79×10−59	[25]
KSPOCP: Ca8H2(PO4)6 ·5H2O	3.71×10−49	[26]
KSPDCPD: CaHPO4·2H2O	2.51×10−7	[22]
KSPβTCP: Ca3(PO4)2	1.22×10−29	[27]
KSPACP: Ca3(PO4)2	5.89×10−26	[28]

**Table 4:** Rate of demineralization obtained from the experimental bench-scale model and the computer simulation of caries

Rate of demineralization of OHAp (μg OHAp/cm^2^ min) “Plaque” pH						
Membrane	3.2	3.5	4.0	4.5	5.0	
*Non-selective*						
Simulation^a^	265.3	105.1	28.11	7.54	2.66	
Experimental^b^	10.4	3.08	0.88	0.28	0.10	
Ratio^c^	25.5	34.1	31.9	26.9	26.6	avg = 29.0
*Cationic*						
Simulation	683.7	294.7	79.64	22.48	6.42	
Experimental	15.7	8.08	2.31	0.62	0.10	
Ratio	43.5	36.5	34.5	36.3	64.2	avg = 43.0
*Anionic*						
Simulation	0.17	0.14	0.09	0.06	0.04	
Experimental	0.19	0.14	0.07	0.03	0.00	
Ratio	0.89	1.00	1.29	2.00		avg = 1.30

a Calculated from the flux of calcium or phosphate from the computer simulation.

b Experimental bench-scale data from [11].

c Ratio of the simulation and experimental flux values.

## 5. Appendix A

Figure 2 shows the solubility isotherms of OHAp expressed as the logarithms of the Ca and P concentrations of a series of saturated solutions. The curve labeled [0] (Insert) represents the isotherm in the 3-component system, Ca(OH)2-H_3_PO_4_-H_2_O, where no “foreign” (noncalcium, non-phosphate) ions are present. *U*(−) [34] represents the concentration of a foreign acidic component such as Cl^−^. Similarly *U*(+) denotes the concentration of Na^+^ if NaOH is a foreign component. Thus the label “−[3]” (“ + [3]”) is to be interpreted as the sum of the dissociated foreign acid (base) ions and charged ion-pairs at a concentration of 10^−3^ eq/L, i.e., p*U*(−) = 3. If several foreign components *U*(±) = |Σ*_i_z_i_C_i_*| represents a net “fourth component,” where *C_i_* is the concentration of the *i*th “foreign” ion or ion-pair, *z_i_* is its valence, and p*U*(±) =−log*U*(±). *U*(+) denotes an excess of foreign base over acid in the summation and contrariwise for *U*(−). Each curve in Fig. 2 represents the applicable solubility of OHAp for a given *pU* value.
